# Ulp1 Regulates Cell Proliferation Through INO1 in *Pichia pastoris*

**DOI:** 10.3390/genes15111459

**Published:** 2024-11-13

**Authors:** Junjie Yang, Bo Zhong, Lan Yang, Zhan Luo, Lei Jia, Kaixi Zheng, Wenjie Tang, Wenna Shang, Xiaofeng Jiang, Zhengbing Lyu, Qijing Gai, Jianqing Chen, Guodong Chen

**Affiliations:** 1 College of Life Sciences and Medicine, Zhejiang Provincial Key Laboratory of Silkworm Bioreactor and Biomedicine, Zhejiang Sci-Tech University, Hangzhou 310018, China; yjj17865567201@163.com (J.Y.); 17367100668@163.com (B.Z.); yangl070916@gmail.com (L.Y.); luo2674@outlook.com (Z.L.); jia2639040474@163.com (L.J.); 17788589466@163.com (K.Z.); 19884162283@163.com (W.T.); 13011786760@163.com (W.S.); xfjiang@zstu.edu.cn (X.J.); zhengbingl@zstu.edu.cn (Z.L.); 2 Zhejiang Sci-Tech University Shaoxing Academy of Biomedicine Co., Ltd., Shaoxing 312369, China; 3 Zhejiang Q-Peptide Biotechnology Co., Ltd., Shaoxing 312366, China; gqjcjq@126.com; 4 School of Life Sciences, Central South University, Changsha 410031, China

**Keywords:** Ulp1, INO1, *Pichia pastoris*, cell cycle, inositol

## Abstract

Background/Objectives: Ulp1 is a vital regulator of the cell cycle, with its absence leading to G2/M phase arrest in *Saccharomyces cerevisiae*. This study aims to investigate the role of Ulp1 in cell cycle regulation in *Pichia pastoris* and to elucidate its mechanisms of action, particularly through the modulation of the gene *INO1*. Methods: We generated Ulp1 knockout strains in *Pichia pastoris* using the FLP-FRT system and performed RNA sequencing (RNA-seq) to analyze gene expression changes. We assessed cell proliferation in *Ulp1* knockout and *INO1* overexpressing strains, as well as the effects of inositol supplementation. Results: Our findings revealed significant downregulation of *INO1* and other genes in *Ulp1* knockout strains. Importantly, overexpression of *INO1* restored cell proliferation, indicating that Ulp1 regulates this process via INO1. Notably, supplementation with exogenous inositol did not rescue cell proliferation, suggesting that the enzymatic activity of INO1 is not required for Ulp1’s regulatory function. Conclusions: This study demonstrates that Ulp1 modulates cell proliferation in *Pichia pastoris* through INO1, independent of its enzymatic activity. These insights enhance our understanding of Ulp1’s role in cell cycle regulation and open new avenues for exploring the molecular mechanisms governing yeast cell division. Further investigations are warranted to delineate the intricate regulatory pathways involved in this process.

## 1. Introduction

The small ubiquitin-like modifier (SUMO) pathway plays a crucial role in various cellular processes, including cell cycle regulation, transcription, and protein trafficking [1,2]. SUMO-specific proteases, such as Ulp1 (ubiquitin-specific protease-like 1), are enzymes that cleave SUMO from SUMO-conjugated proteins, thereby reversing SUMO modifications [3,4]. In *S. cerevisiae*, Ulp1 has been extensively studied and found to be essential for cell viability, as its absence leads to G2/M cell cycle arrest [5,6]. Similarly, in the methylotrophic yeast *P. pastoris*, Ulp1 has also been identified as a key regulator of the cell cycle. *P. pastoris* is a widely used host for the production of recombinant proteins due to its efficient secretion system [7,8,9]. However, the functions of Ulp1 in *P. pastoris* have not been extensively explored. Understanding the role of Ulp1 in *P. pastoris* cell cycle regulation could provide valuable insights into improving recombinant protein production in this yeast.

The cell cycle is a highly regulated process that ensures the accurate replication and segregation of genetic material into daughter cells. In eukaryotes, the cell cycle is composed of four main phases: G1 (gap 1), S (DNA synthesis), G2 (gap 2), and M (mitosis) [10]. Progression through the cell cycle is controlled by a complex network of checkpoint mechanisms and regulatory proteins, such as cyclins and cyclin-dependent kinases (CDKs) [11,12,13]. In yeast, the cell cycle is similarly regulated, and various studies have identified key players in this process, including the SUMO pathway and its associated enzymes, i.e., Ulp1 [5,14,15].

In this study, we investigated the functions of Ulp1 in *P. pastoris* and its impact on cell cycle regulation. We identified three Ulp1-like proteins in *P. pastoris* and focused on the role of the pUlp1 homolog, which is the functional equivalent of Ulp1 in *S. cerevisiae*. By generating *Ulp1* knockout strains in *P. pastoris*, we observed G2 phase stalling, similar to the phenotype reported in *S. cerevisiae* [5]. Additionally, our transcriptome analysis revealed significant changes in the expression of several genes, including *INO1*, which is involved in inositol biosynthesis. Interestingly, the overexpression of *INO1* in *Ulp1* knockout cells restored cell proliferation, indicating that Ulp1 regulates cell proliferation by modulating *INO1* expression. This suggests that *INO1* is the primary target of the Ulp1-Scs2-Siz2 pathway involved in the exit frommitosis [16,17,18]. These findings provide new insights into the role of Ulp1 in the cell cycle and raise intriguing questions about the complex regulatory mechanisms governing yeast cell division.

## 2. Materials and Methods

### 2.1. Identification of Ubiquitin-like Specific Proteases in P. pastoris

The Ulp1 sequence from *S. cerevisiae* (Uniprot ID: Q02724) was downloaded and used as a query in a tblastn search to identify ubiquitin-like specific proteases in the *P. pastoris (Komagataella pastoris)* NCBI genome database. We identified three Ulp1-like protein coding genes, which we named pUlp1, pUlp2, and pUlp3, based on their sequence similarity to the Ulp1 protein from *S. cerevisiae*.

### 2.2. Construction of Specific P. pastoris Strains

The pPICZ-FLP-DouH5, pPICZ-FLP-DouH3, pPICZ-FLP-DouH3a, pUFRT-GFP-Avi, and pUFRT-GFP-Avi-INO1 plasmids were constructed according to the instructions provided in the Seamless Cloning Kit (Beyotime (Shanghai, China), D7010M). The strains obtained and used are shown in Table 1. Newly prepared yeast competent cells were created using 1 mol/L sorbitol. The plasmids were linearized with appropriate primer pairs and transformed into yeast competent cells using the Bio-Rad (Hercules, CA, USA) MicroPulser, Fungi2-Pic, at 2 kV. The cells were then cultured at 28 °C for 2–3 days. The clones were selected and validated by PCR.

**Figure 1:**
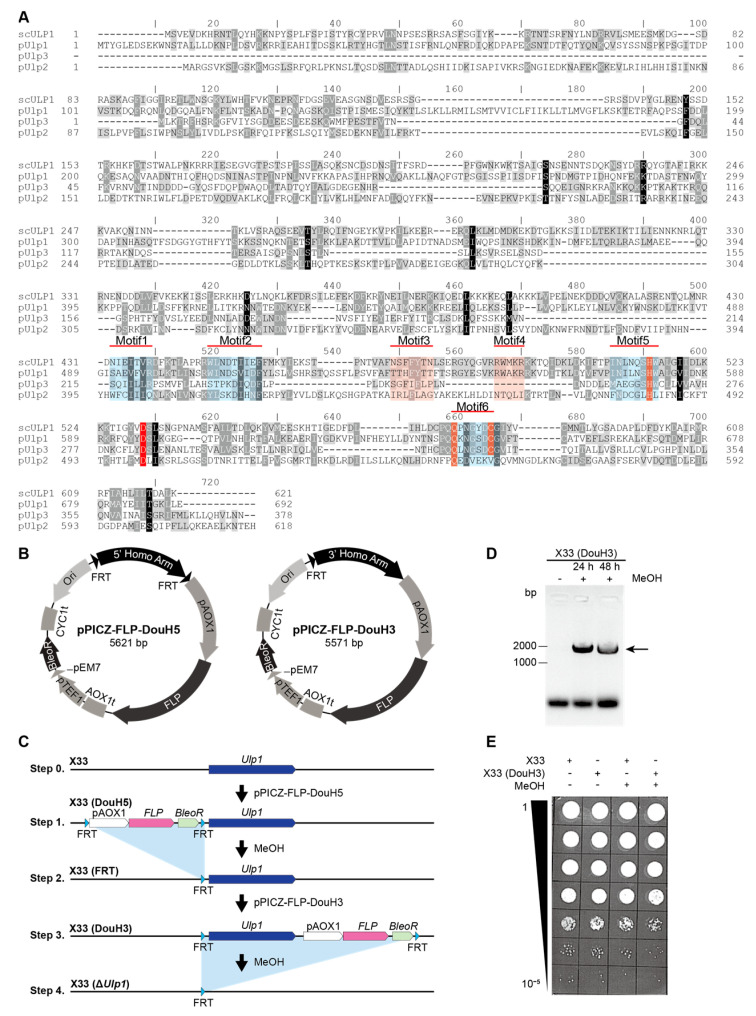
*Ulp1* knockout using the two-plasmid strategy. (**A**) Amino acid sequence alignment of scUlp1, pUlp1, pUlp2, and pUlp3. (**B**) Plasmids for *Ulp1* knockout. DouH5 is used for FRT insertion upstream of *Ulp1*, and DouH3 is used for *Ulp1* ablation with methanol induction. (**C**) Strategy of Ulp1 knockout. The FLP and FRT system is used for *Ulp1* knockout *P. pastoris* construction. (**D**) Genotyping of *Ulp1* knockout *P. pastoris* strain. *Ulp1* knockout *P. pastoris* is detected via PCR after methanol induction. (**E**) Growth test of X33 (wild type) and X33 (DouH3) strains. The growth of the X33 (DouH3) strain is decreased compared to that of the X33 strain under methanol induction.

**Figure 2:**
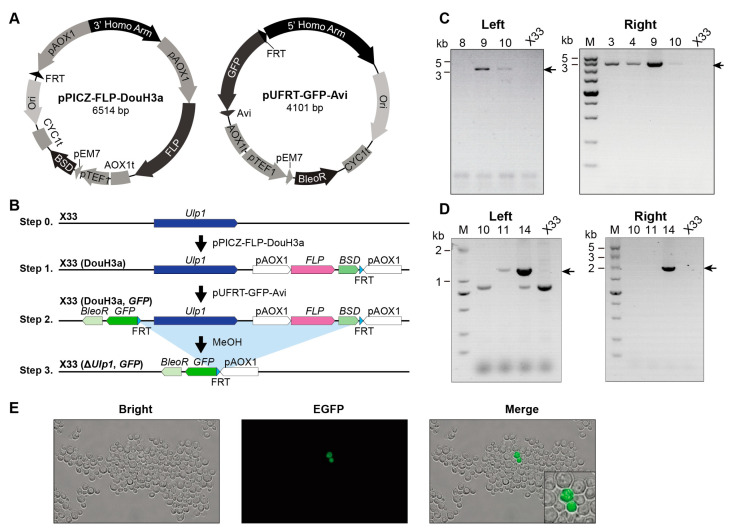
*Ulp1* knockout *P. pastoris* can bud, but the buds fail to detach from the mother cell. (**A**) Plasmids used for constructing the inducible *Ulp1* knockout *P. pastoris* strain with the reporter gene *GFP.* Plasmid DouH3a is used for FLP and 3′ FRT insertion, and pUFRT-GFP-Avi is employed for the reporter gene *GFP* and 5′ FRT insertion. Following the deletion of *Ulp1*, GFP expression is induced. (**B**) Strategy for constructing the inducible *Ulp1* knockout *P. pastoris* strain with the reporter gene *GFP*. (**C**) X33 (DouH3a) *P. pastoris* clone genotyping. The arrow represents the size of the destination strip. (**D**) X33 (DouH3a, GFP) *P. pastoris* clone genotyping. The arrow represents the size of the destination strip. (**E**) Fluorescent microscopic images of the inducible *Ulp1* knockout *P. pastoris* strain reveal detectable green fluorescence in the *Ulp1* knockout cells. The cells are capable of normal budding; however, the buds do not detach from the mother cell.

**Figure 3:**
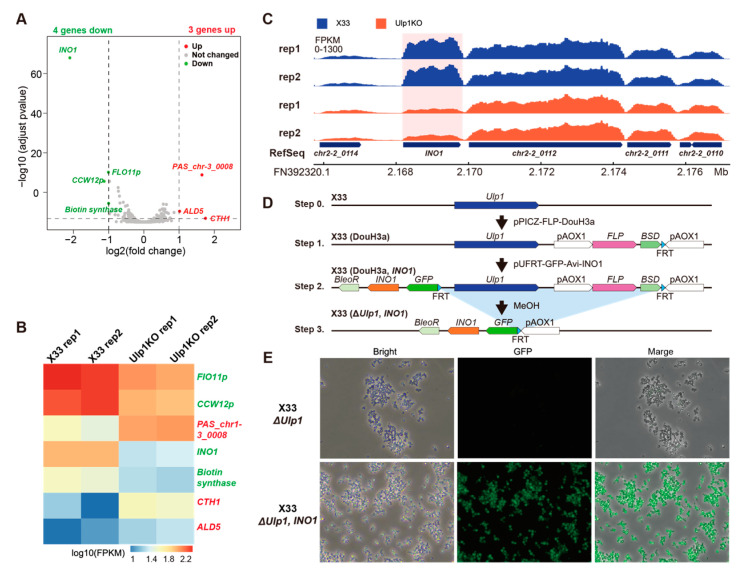
Overexpression of *INO1* in *Ulp1* knockout *P. pastoris* rescues phenotypes associated with cell cycle arrest. (**A**) Volcano plot of RNA-seq data comparing X33 and *Ulp1* knockout *P. pastoris* cells. Four genes were downregulated, and three genes were upregulated. Red indicates upregulated genes, green denotes downregulated genes, and gray represents genes that are not significantly changed. Upregulated genes are defined by log_2_(fold change) ≥ 1 and padj ≤ 0.05, while downregulated genes are defined by log_2_(fold change) ≤ −1 and padj ≤ 0.05, where padj represents the adjusted *p*-value. The Benjamini–Hochberg method is used for differential expression analysis. Threshold: |log_2_(Fold Change)| ≥ 1 and adjust *p*-value ≤ 0.05. (**B**) Heatmap of significantly changed genes in Ulp1 knockout cells versus X33 cells. FPKM (fragments per kilobase of transcript per million mapped reads) is a normalization method used in RNA-seq analysis to account for the effects of sequencing depth and gene length. FPKM = mumber of fragments mapped to a gene × 10^9^/(total number of mapped fragments × length of the gene in kilobases). (**C**) RNA-seq tracks for the *INO1* locus. *INO1* was significantly downregulated in *Ulp1* knockout cells compared to X33 cells. (**D**) Workflow of the *INO1* rescue experiment. Extra *INO1* was inserted into the *Ulp1* locus, and the expression of *INO1* was controlled by the *AOX1* promoter. (**E**) Phenotypes of *Ulp1* knockout cells, without (X33 Δ*Ulp1*) and with (X33 Δ*Ulp1*, *INO1*) extra *INO1* expression. Extra *INO1* expression in *Ulp1* knockout cells restored normal cell proliferation.

For the FLP inducible cutting steps [19], the cells were cultured in glycerol medium (BMGY) for 24 h, then transferred to 1% methanol medium (BMMY) and induced for 48–72 h, with extra methanol added to the culture every 24 h. The strategies are outlined in Figure 1C, Figure 2B and Figure 3D.

### 2.3. Library Preparation for Transcriptome Sequencing

Total RNA was used as the input material for the RNA sample preparations. Briefly, mRNA was purified from total RNA using poly-T oligo-attached magnetic beads. Fragmentation was carried out using divalent cations under elevated temperature in First Strand Synthesis Reaction Buffer (5×). First strand cDNA was synthesized using random hexamer primers and M-MuLV Reverse Transcriptase (RNase H−). Second strand cDNA synthesis was subsequently performed using DNA Polymerase I and RNase H. Remaining overhangs were converted into blunt ends via exonuclease/polymerase activities. After adenylation of the 3′ ends of DNA fragments, adaptors with hairpin loop structures were ligated to prepare for hybridization.

To preferentially select cDNA fragments measuring 370–420 bp in length, the library fragments were purified using the AMPure XP system. PCR was then performed with Phusion High-Fidelity DNA Polymerase, Universal PCR primers, and Index (X) Primer. Finally, PCR products were purified using the AMPure XP system, and library quality was assessed via the Agilent Bioanalyzer 2100 system.

### 2.4. RNA-Seq Data Analysis

Raw data (raw reads) in the FASTQ format were first processed using fastp software v0.23.2. During this step, clean data (clean reads) were obtained by removing reads containing adapters, reads containing poly-N, and low-quality reads from the raw data. Additionally, the Q20, Q30, and GC content of the clean data were calculated. All downstream analyses were based on the high-quality clean data. The reference genome (NCBI accession number: GCA_000027005.1_ASM2700v1) and the corresponding gene annotation files were downloaded directly from the NCBI website. The index for the reference genome was constructed using Hisat2, with default parameters. Paired-end clean reads were aligned to the reference genome in paired-end mode using Hisat2 [20]. Gene read counts were calculated using HTSeq, and differentially expressed genes were identified using DESeq2. For differential expression analysis, the thresholds were set to |log2Fold Change| ≥ 1 and an adjusted *p*-value ≤ 0.05.

## 3. Results

### 3.1. Ulp1 Is Essential for P. pastoris Proliferation

Several laboratories have reported the essential role of Ulp1 in the mitosis of *S. cerevisiae* [5]. However, there is little research on the functions of Ulp1 in *P. pastoris*. In this study, we performed a tblastn search using the Ulp1 protein sequence of *S. cerevisiae* (scUlp1) to identify homologs in *P. pastoris* [21]. We identified three Ulp1-like proteins, which we designated as pUlp1, pUlp2, and pUlp3 (Figure 1A), and we found six conserved motifs in the homologs of scUlp1 and pUlp1, indicating that pUlp1 is the functional equivalent of scUlp1 in *P. pastoris* (Figure 1A).

Ulp1 is an enzyme responsible for cleaving SUMO/Smt3 tags from SUMO-tagged proteins, which prevents the secretion of SUMO-tagged foreign proteins from yeast [22]. Based on this knowledge, we decided to delete *pUlp1* in *P. pastoris*. In the subsequent sections of this article, we will refer to *pUlp1* as *Ulp1*. To replace *Ulp1*, we performed a double crossover using BleoR and FLP. However, we were unable to obtain any BleoR-positive clones. To address the possibility of low recombination efficiency, we performed two single-crossover assays to eliminate *Ulp1* (Figure 1B,C). PCR results demonstrate that *Ulp1* can be eliminated following methanol induction. However, we were unable to achieve any *Ulp1* knockout clones (BleoR negative) (Figure 1D,E), indicating that *Ulp1* is essential for *P. pastoris*.

### 3.2. The Recombination Rate of FLP Is Decreased in Ulp1 Conditional Knockout P. pastoris

We observed a slight decrease in the number of X33(DouH3) clones following methanol induction (Figure 1E). To further investigate the viability of yeast cells lacking *Ulp1*, we introduced a reporter gene, GFP, which would be expressed upon *Ulp1* elimination (Figure 2A,B). Using PCR, we confirmed the correctness of the X33(DouH3a) #9 clones (Figure 2C) and the X33(DouH3a, GFP) #14 clones (Figure 2D).

Following methanol induction, GFP expression was observed in only about 2‰ (0.2%) of the clones (Figure 2E). This observation indicates a significant decrease in FLP recombination efficiency in the Ulp1 conditional knockout strain of *P. pastoris*. The reduced number of GFP-expressing clones suggests that while some cells can initiate the knockout process, the complete elimination of Ulp1 severely impacts cell viability and proliferation.

Our findings imply that the complete ablation of *Ulp1* from *P. pastoris* is not feasible under the conditions tested. Although the yeast cells lacking *Ulp1* can survive, to some extent, they are unable to undergo successful cell division. This inability to proliferate might be attributed to the essential role of Ulp1 in the cell cycle, particularly in transitioning through the G2 phase [5,23].

### 3.3. Overexpression of INO1 Rescues Cell Proliferation in Ulp1 Knockout P. pastoris

Ulp1 is essential for yeast cell cycles, although the underlying mechanism remains unknown. To uncover this mechanism, we performed RNA-seq on the X33 (WT) and *Ulp1* knockout yeast strains. RNA-seq data showed that the expression of four genes, including INO1, was downregulated, consistent with previous reports for *S. cerevisiae* [17], and three genes were upregulated (Figure 3A–C). *INO1* encodes the inositol-3-phosphate synthase enzyme, which catalyzes the rate-limiting step in the biosynthesis of inositol. Deletion of *INO1* results in inositol auxotrophy, meaning that the yeast cells require exogenous inositol for growth and survival. Whether or not Ulp1 regulates cell proliferation through *INO1* expression is still unknown. To address this question, we constructed the *INO1* overexpressed *P. pastoris* strain X33 (DouH3a, *INO1*), in which *Ulp1* knockout induces GFP expression, and the extra *INO1* expression is controlled by the AOX1 promoter (Figure 3D). To our surprise, we observed that *Ulp1* knockout cells restored cell proliferation in the X33 (Δ*Ulp1*, *INO1*) strain (Figure 3E). These data suggest that INO1 can rescue the cell proliferation phenotype of *Ulp1* knockout cells, indicating that Ulp1 regulates cell proliferation through INO1.

### 3.4. The Addition of Extra Inositol and Fe^2+^ Does Not Rescue Cell Proliferation in Ulp1 Knockout P. pastoris

**Figure 4:**
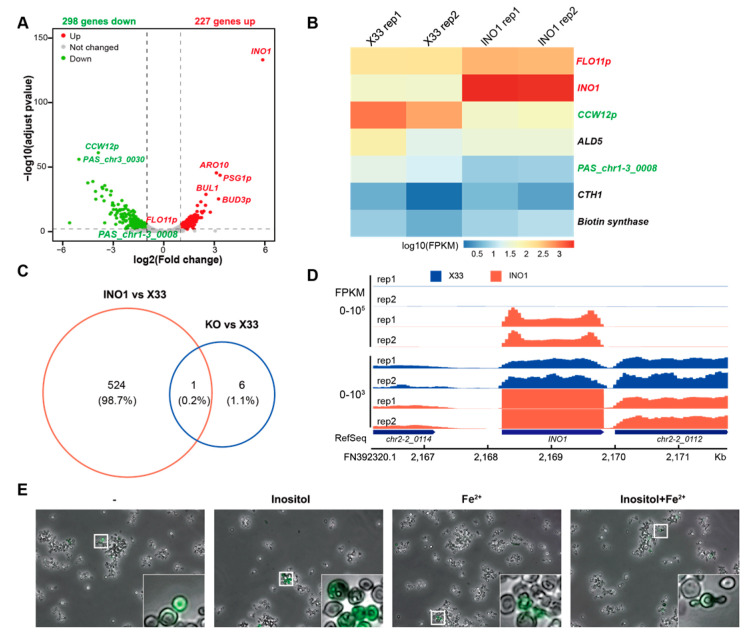
Adding extra inositol and/or Fe^2+^ is unable to rescue *Ulp1* knockout cell phenotypes. (**A**) Volcano plot of RNA-seq data comparing X33 and *INO1*-overexpressing Ulp1 knockout *P. pastoris* cells. A total of 298 genes were downregulated, and 227 genes were upregulated. Red denotes upregulated genes, green indicates downregulated genes, and gray represents genes with no significant change. Upregulated genes are defined as having log2(fold change) ≥ 1 and padj ≤ 0.05, while downregulated genes have log2(fold change) ≤ −1 and padj ≤ 0.05, where padj refers to the adjusted *p*-value. (**B**) Heatmap of significantly changed genes in *INO1*-overexpressing *Ulp1* knockout cells versus X33 cells. (**C**) Venn diagram of significantly changed genes between *INO1*-overexpressing *Ulp1* knockout cells versus X33 cells and *Ulp1* knockout cells versus X33 cells. Only the expression of *CCW12p* was not restored. The red circle represents significantly changed genes in the *INO1*-overexpressing *Ulp1* knockout cells compared to those of the wild-type strain X33, while the blue circle indicates significantly changed genes in the *Ulp1* knockout cells relative to those of the wild-type strain X33. (**D**) RNA-seq tracks for the *INO1* locus. *INO1* under the *AOX1* promoter was upregulated more than 100-fold compared to the X33 cells. (**E**) Phenotypes of *Ulp1* knockout cells with the addition of nothing (−), inositol (Inositol), Fe^2+^ (Fe^2+^), and inositol and Fe^2+^ (Inositol + Fe^2+^). The addition of inositol and/or Fe^2+^ did not rescue *Ulp1* knockout cells back to normal cell proliferation.

As mentioned previously, INO1 catalyzes the rate-limiting step in the biosynthesis of inositol. To understand the mechanism by which INO1 regulates *P. pastoris* cell proliferation, we employed RNA-seq on the X33 (WT) and X33 (Δ*Ulp1*, *INO1*) strains. RNA-seq data showed that the expression of 298 genes was downregulated, and 227 genes, including *INO1*, were upregulated (Figure 4A). We found that the expression of six genes, except *CCW12p*, was rescued when we overexpressed *INO1* in *Ulp1* knockout cells (Figure 4B,C). Since the *AOX1* promoter is a strong inducible promoter, we observed that *INO1* was upregulated more than 100-fold compared to that of X33 (Figure 4D). Among these rescued genes, we identified *CTH1*. *CTH1* encodes an RNA-binding protein that regulates the expression of iron-responsive genes [24,25]. The targets of CTH1 include genes involved in iron-containing proteins, iron uptake, and iron utilization pathways. By downregulating these iron-dependent genes, CTH1 helps the cell prioritize the use of limited iron resources during times of iron scarcity. To further investigate this process, we added extra inositol and Fe^2+^ to the *Ulp1* knockout cells. We observed that additional inositol, Fe^2+^, and the combination of inositol and Fe^2+^ were not able to rescue the phenotype of the *Ulp1* knockout cells (Figure 4E). These data suggest that INO1 regulates cell proliferation independently through the inositol synthesis pathway.

## 4. Discussion

For more than two decades, *Ulp1* has been reported to regulate cell cycles in yeast, but the mechanisms of how Ulp1 regulates cell cycle progression are still not well understood. Previous research has shown that Ulp1-mediated deSUMOylation is a critical regulatory mechanism that controls multiple key transitions and events throughout the yeast cell cycle, from the G2/M phase transition to mitotic spindle assembly and mitotic exit [22,26]. The interactions and localization of proteins have a significant impact on the cell cycle [27]. Ulp1 ensures the proper execution of processes such as DNA synthesis by regulating associated nuclear pore complexes [28]. Ref. [16] explored the effects of SUMOylation and deSUMOylation on the localization of INO1 to the nuclear pore complex during the late mitotic stage and the G1 phase. Ulp1 regulates INO1 expression and cellular localization by modulating SUMOylation and deSUMOylation, thereby influencing yeast cell proliferation [18].

While extensive research has focused on Ulp1 in *S. cerevisiae*, comparative studies of Ulp1 homologs across different organisms have revealed both conserved and divergent roles. In *Schizosaccharomyces pombe*, the Ulp1 homolog is involved in the regulation of the cell cycle and the DNA damage response, emphasizing its importance in maintaining genomic stability [29]. In *Drosophila melanogaster*, Ulp1 homologs have been shown to play roles in development and apoptosis, highlighting their involvement in critical processes beyond cell division [30]. In mammals, the Ulp1 homolog SENP1 is implicated in regulating stem cell differentiation, response to hypoxia, and tumorigenesis, suggesting a more complex role in cellular adaptation and survival [31,32]. In our study, we present evidence that Ulp1 regulates the cell cycle, primarily by controlling the expression of *INO1* in *P. pastoris*. Our findings reveal that the overexpression of *INO1* in *Ulp1*-deficient cells effectively restores normal cell proliferation, indicating that *INO1* is a crucial target for Ulp1 in the regulation of cell cycle progression. This highlights the significant role of INO1 not only as a metabolic enzyme involved in inositol synthesis but also as a key regulator of cell cycle dynamics. The ability of INO1 to rescue the proliferation defect in *Ulp1*-deficient backgrounds underscores its importance in maintaining cellular function and suggests that Ulp1 may influence the cell cycle through an inositol-independent pathway. These insights contribute to a deeper understanding of the molecular mechanisms by which Ulp1 modulates cell cycle regulation in *P. pastoris*, paving the way for future investigations into the specific pathways and interactions involved.

INO1 is essential for inositol synthesis, catalyzing the conversion of glucose-6-phosphate to inositol-1-phosphate, which ultimately produces inositol [33]. This process is not only a critical step in inositol synthesis but also a fundamental mechanism for regulating intracellular inositol levels. Inositol plays a vital role in cell proliferation [34], serving as a precursor for cellular membrane components [35], participating in phospholipid synthesis [36], and acting as a second messenger in signal transduction [37,38,39]. It is involved in regulating various cellular processes, including the cell cycle, apoptosis, and stress responses [40,41,42]. The absence of INO1 results in inositol auxotrophy [43]. To further elucidate the underlying mechanisms, we investigated how INO1 regulates the cell cycle. Initially, we hypothesized that the cell cycle arrest observed in *Ulp1* knockout cells was due to inositol depletion. However, to our surprise, overexpressing *INO1* could rescue the cell cycle arrest phenotype, while the addition of exogenous inositol was unable to do so. Additionally, we found that the major myo-inositol transporter Itr1p is present in *P. pastoris*, suggesting that INO1 regulates the cell cycle through an inositol-independent pathway. Further investigations are necessary to uncover the precise mechanisms by which INO1 modulates cell cycle progression.

Another important question is whether the deSUMOylation of proteins other than those regulating *INO1* expression, such as Scs2 [17], is essential for cell cycle control. In *P. pastoris*, the overexpression of *INO1* could rescue the cell cycle arrest phenotype, suggesting that the deSUMOylation of key cell cycle regulators, such as the mitotic kinase Cdc28/Cdk1 [44,45], may not be essential. However, we cannot exclude the possibility that INO1 may serve as a bridge to recruit Ulp1, Ulp2, or Ulp3 to target proteins, thereby facilitating their deSUMOylation. Additional experiments are required to determine the significance of the deSUMOylation of specific cell cycle regulators.

Furthermore, while some studies in *S. cerevisiae* have reported that inositol can rescue the phenotypes of *INO1* knockout cells [46,47], our findings in *P. pastoris* indicate that inositol is unable to rescue *INO1* knockdown cells. This suggests that the functions of Ulp1 in *P. pastoris* may differ from those in *S. cerevisiae*, and that Ulp1 may regulate the cell cycle through different mechanisms in these two yeast species. Further comparative studies between these species are necessary to elucidate these differences.

In summary, our study demonstrates that Ulp1 regulates cell proliferation through *INO1* in *P. pastoris*, offering new insights into the role of Ulp1 in cell cycle regulation. This finding not only deepens our understanding of the molecular mechanisms governing yeast cell proliferation but also has broader implications for biotechnological applications, such as the optimization of yeast strains for industrial processes. Furthermore, this discovery opens up new avenues for exploring the diverse regulatory mechanisms that yeast cells employ to control their cell cycles, highlighting the potential for future research to uncover additional layers of complexity in this vital biological process.

## Figures and Tables

**Table 1:** Strains and genotypes.

Strain	Genotypes	Purpose
X33	-	Initial strain
X33(DouH5)	*FLP*, *BleoR*, 5′ FRT	Introduce FRT sites at the 5′ end of *Ulp1*
X33(FRT)	5′ FRT	Ablate *BleoR* and *FLP* from the X33 (DouH5) strain, and keep one FRT site
X33(DouH3)	*FLP*, *BleoR*, 5′ FRT, 3′ FRT	Introduce *FLP* and another FRT site at the 3′ end of *Ulp1*
X33(Δ*Ulp1*)	*Ulp1*Δ	Use the FLP/FRT knockout system to knock out *Ulp1* from the genome; *BleoR* and *FLP* were also ablated
X33(DouH3a)	*FLP*, *BSD*, 3′ FRT	Introduce the FLP enzyme, one FRT site, and the *AOX* promoter at the 3′ end of *Ulp1*
X33(DouH3a, *GFP*)	*FLP*, *BSD*, 3′ FRT, *GFP*, *BleoR*	Introduce one FRT site at the 5′ end of *Ulp1* and a *GFP* that can be expressed after knocking out *Ulp1*
X33(Δ*Ulp1*, *GFP*)	*Ulp1*Δ, *GFP*, *BleoR*	Obtain *Ulp1* knockout strains containing the reporter gene *GFP*
X33(DouH3a, *INO1*)	*FLP*, *BSD*, 3′ FRT, *GFP*, *INO1*, *BleoR*	Introduce the FRT site, *GFP*, and *INO1* at the 5′ end of *Ulp1*
X33(Δ*Ulp1*, *INO1*)	*Ulp1*Δ, *GFP*, *INO1*, *BleoR*	Overexpress *INO1* during *Ulp1* knockout

## Data Availability

The data that support the findings of this study are available from the corresponding author upon reasonable request. The raw data are available in NCBI GEO: GSE274307.
